# Inflammation markers in the saliva of infants born from Zika-infected mothers: exploring potential mechanisms of microcephaly during fetal development

**DOI:** 10.1038/s41598-019-49796-5

**Published:** 2019-09-20

**Authors:** Diogo N. de Oliveira, Estela O. Lima, Carlos F. O. R. Melo, Jeany Delafiori, Tatiane M. Guerreiro, Rafael G. M. Rodrigues, Karen N. Morishita, Cynthia Silveira, Stéfanie Primon Muraro, Gabriela Fabiano de Souza, Aline Vieira, Antônio Silva, Rosângela F. Batista, Maria J. R. Doriqui, Patricia S. Sousa, Guilherme P. Milanez, José L. Proença-Módena, Denise P. Cavalcanti, Rodrigo R. Catharino

**Affiliations:** 1 Innovare Biomarkers Laboratory, School of Pharmaceutical Sciences, University of Campinas, Campinas, Brazil; 2 Medical Genetics Department, School of Medical Sciences, University of Campinas, Campinas, Brazil; 3 Emerging Viruses Study Laboratory, Department of Genetics, Evolution, Microbiology and Immunology, Biology Institute, University of Campinas, Campinas, Brazil; 4 Public Health Department, Universidade Federal do Maranhão, São Luís, Brazil

**Keywords:** Viral host response, Biomarkers

## Abstract

Zika virus (ZIKV) has emerged as one of the most medically relevant viral infections of the past decades; the devastating effects of this virus over the developing brain are a major matter of concern during pregnancy. Although the connection with congenital malformations are well documented, the mechanisms by which ZIKV reach the central nervous system (CNS) and the causes of impaired cortical growth in affected fetuses need to be better addressed. We performed a non-invasive, metabolomics-based screening of saliva from infants with congenital Zika syndrome (CZS), born from mothers that were infected with ZIKV during pregnancy. We were able to identify three biomarkers that suggest that this population suffered from an important inflammatory process; with the detection of mediators associated with glial activation, we propose that microcephaly is a product of immune response to the virus, as well as excitotoxicity mechanisms, which remain ongoing even after birth.

## Introduction

With the recent outbreak of ZIKV infection in Brazil in 2015 and 2016, followed by the intimate association between this infection and the increase in microcephaly, well-documented by case reports, public health authorities throughout the country assumed a state of alert that impacted the worldwide health community^1,2^. The neurotropism of ZIKV in adults and children has been studied in the last years, and viral pathogenesis is known to lead to autoimmune aggravations such as the Guillain-Barré syndrome^3,4^. Effects on infected pregnant women are even more devastating, as ZIKV is able to cross the placental and blood-brain barriers, causing disruptions in the developing brain of the fetus, impairing the growth of neural progenitor cells (NPCs), which ultimately lead to brain damage^5–7^. Furthermore, the role of ZIKV as a teratogen is strongly implicated from an epidemiological point of view, and the phenotypic spectrum of CZS has been accurately described and outlined by neuroimaging techniques^8,9^.

Studies detailing the pathophysiological effects and the cell machinery involved in ZIKV infection and proliferation are providing clearer information on the viral replication cycle, as research advances towards effective screening methods for viral detection in human fluids^10^. The mechanisms leading to neural underdevelopment in human NPCs, nonetheless, remain little known^11^. Recent literature reports rely on the hypothesis that ZIKV promotes impaired NPCs growth, with associated apoptotic events, exerting similar effects over the systems of both newborns and adults^12^. The idea of interfering in the replication of cells with high metabolic rate has even elicited a whole new line of studies where ZIKV was used in an oncolytic-like strategy to treat aggressive gliomas, with results showing important cytopathic effects of the virus over the tumors^13,14^.

The mechanisms by which flaviviruses migrate to the CNS, i.e. neurotropism, are still unresolved. Current literature describes three possible migration pathways: (i) through peripheral nerves after the mosquito bite, a mechanism that involves retrograde transportation through axons; (ii) through the bloodstream, crossing the blood-brain barrier (BBB) with altered permeability resulting from the presence of pro-inflammatory species; and (iii) carried by immune cells, a mechanism also known as ‘Trojan horse’ neuroinvasion^15–18^. Given this particular migratory behavior, and the trend to investigate the traces and effects of ZIKV infection using noninvasive biofluids, we hereby report the identification of three inflammatory mediators that were detected in the saliva of microcephalic infants after birth, all them from mothers that were diagnosed with Zika infection during pregnancy. Suggesting that CZS subjects overexpress inflammatory biomarkers in saliva is consistent with a status of ongoing neurological inflammation, and may be supported by the anatomical relationship of salivary glands and developing nerves, such as the trigeminal nerve^19^. Furthermore, our findings also support the utility of saliva sampling to monitor the status of this disease, corroborating the utility of this biofluid as a non-invasive approach for biomarker screening purposes, as recently demonstrated by Huan *et*. *al* in a contribution with Alzheimer’s Disease^20^.

Our hypothesis, therefore, is that the pathways associated with the biomarkers described are reflective of the anti-ZIKV infection cellular immune response, and that they may be involved with the neural damage that leads to microcephaly.

## Metabolomics to Unravel Microcephaly-Related Biomarkers

**Figure 1:**
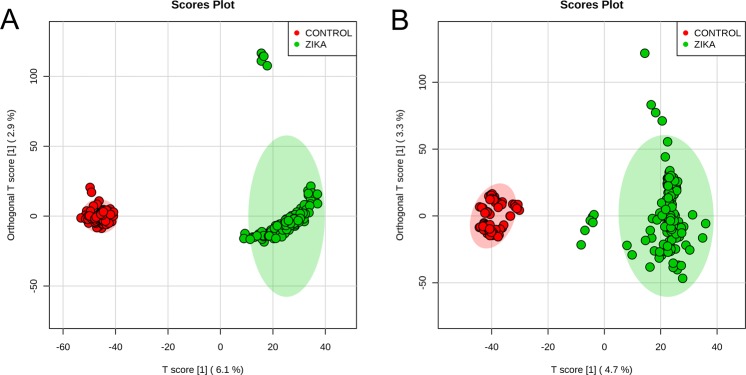
OPLS-DA score plots indicating clear separation between the group of infants with congenital zika syndrome (green) and the control group (red). The statistical model was able to distinguish the high-resolution mass spectrometry-generated datasets from both the positive (**A**) and negative (**B**) ion modes, in a strong indication that the chemical composition of the two groups is different.

A statistical model using orthogonal partial least squares discriminant analysis (OPLS-DA) was applied to a high-resolution mass spectrometric dataset from saliva samples, assessing the differences between the metabolomic profile of two distinct groups of infants (n = 27 each): babies diagnosed with CZS upon birth, and healthy subjects with no clinical diagnosis of CZS. From the scores plot in Fig. 1, it is possible to visually assess the difference between groups in both ion modes, where control subjects and microcephalic patients cluster clearly differently in both positive and negative ion modes. Particularities of each group were addressed by a list of significant features generated by the variable importance in projection (VIP) scores from OPLS-DA, which provided a collection of 28 ions that are specific for the congenital microcephaly group. Out of the 28 features selected by OPLS-DA, we were able to identify three compound classes that make sense within the biochemical context of compromised neural development upon ZIKV infection.

## Inflammation as an Important Player in Impaired Neural Growth

Neuroinflammation has been proposed as one of the key factors that contribute to ZIKV-related microcephaly^21^, especially inflammatory processes mediated by glial cells. The series of molecules described in Table 1 compose a list of inflammatory mediators that participate directly in mechanisms associated with immune response, excitotoxicity, as well as physiological responses to minimize further damage to neural tissue. Their correlation with neuroinflammation is suggested by a series of seven elected isomeric prostaglandin analogues derived from PGD_2_, PGE_1_, PGE_2_, and PGI_2_. The role of these species and their derivatives in protecting the CNS is widely discussed in the literature, and it is known that they reflect an anti-inflammatory response to neuronal injuries^22^, contributing to neuroprotection in the CNS in the context of excitotoxicity^23^, which is a known contributor to acute brain injury^24^. Recently, Olmo, *et al*.^25^ described the increase in cytokines TNF-α and IL-1β, and glutamate *in vitro*, after infecting neuronal cells with ZIKV, demonstrating excitotoxicity induction as an important mechanism for the neurotoxic effects observed. Additionally, multiplex analyte-specific beads assays in serum samples have demonstrated that the production of immune mediators can modulate the clinical outcome after ZIKV infection, since the levels of IL-22, MCP-1, IP-10, and TNF-α are usually higher in patients with severe disease and neurological impairment^26^. Thus, our findings indicate that these mechanisms may be active in CZS, as the identified collection of prostaglandin metabolites are putatively overexpressed in response to the infection.

**Table 1:** Identified species, elected as biomarkers for the CZS group.

Compound	Experimental Mass	Theoretical Mass	Error	Adduct	METLIN ID
Ethyl-PGE_2_, and/or Dimethyl-PGD_2_, and/or Dimethyl-PGE_2_, and/or Dihomo-PGI_2_, and/or Dihomo-PGD_2_, and/or Didehydro-dimethyl-PGE_1_, and/or Dihomo-PGE_2_	403.2461	403.2455	−1.5	[M + Na]^+^	36221, 36123, 36136, 45950, 36205, 3014 and 36204
Hepoxilin A_3_ and/or B_3_	359.2192	359.2198	1.7	[M + Na]^+^	7070 and 7071
15-deoxy-δ-12,14-PGJ_2_	351.1727	351.1732	1.4	[M+Cl]^−^	36099

**Figure 2:**
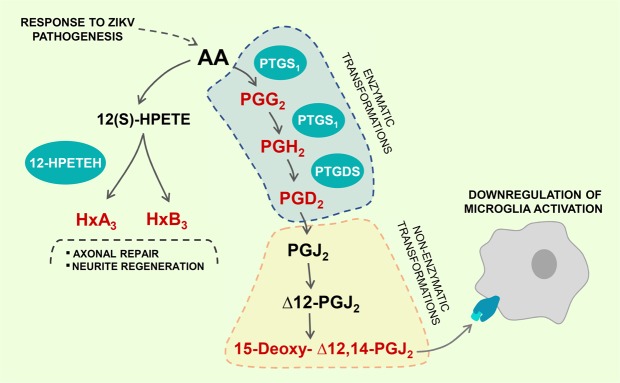
A conception of metabolic pathways that are putatively activated in response to ZIKV pathogenesis, as suggested by the markers from OPLS-DA (in red). Arachidonic acid (AA) is the main precursor activated in this mechanism, with two main cascades unfolding upon activation: prostaglandin metabolism (and its derivatives), and hepoxilin metabolism. All highlighted mediators are linked with response to neural damage and show the organism attempting to attenuate nervous system deterioration. AA: arachidonic acid; 12(S)-HPETE: 12-hydroperoxyicosatetraenoic acid; 12- HPTEH: HxA_3_ and HxB_3_: hepoxilin A_3_ and B_3_; PTGS_1_: prostaglandin-endoperoxide synthase 1 (COX-1); PGG_2_: prostaglandin G_2_; PGH_2_: prostaglandin H_2_; PTGDS: prostaglandin-H_2_ D-isomerase; PGD_2_: prostaglandin D_2_; PGJ_2_: prostaglandin J_2_; Δ12-PGJ_2_: delta-12-prostaglandin J_2_.

The abovementioned activation of the prostaglandin pathway due to excitotoxicity is reinforced by the identification of 15-deoxy-Δ-^12,14^-Prostaglandin J_2_ (15d-PGJ_2_) as a marker for the CZS group. This molecule is a non-enzymatic, end-metabolite of the PGD_2_ pathway, closely linked with microglial activation and neural tissue depletion, as observed in Fig. 2. Furthermore, 15d-PGJ_2_ is the natural agonist of the peroxisome proliferator-activated receptor-γ (PPAR-γ), a nuclear receptor that controls the activation of peroxisomes and, further downstream, negatively regulates microglial function^27^. The identification of 15d-PGJ_2_ as a biomarker for CZS is consistent with enhanced PGD_2_ metabolism, providing a neuroprotective response to reduce cytokine-driven excitotoxicity, as previously reported by Liang *et al*.^23^. The non-enzymatic metabolism of PGD_2_ intermediaries lead to an accumulation of 15d-PGJ_2_, which we propose directly activates PPAR-γ, driving changes of gene expression and eliciting the downregulation of microglial activation in spite of the proinflammatory environment associated with the driving of neural damage^28^.

**Figure 3:**
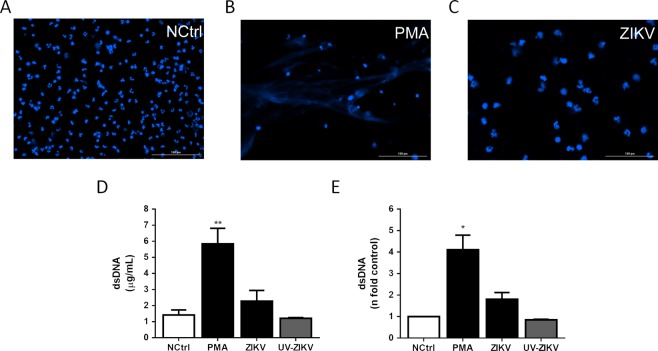
Extracellular DNA quantification from ZIKV-infected human neutrophils. (**A**–**C**) Human neutrophils (1 × 10^5^/300 μL) were stimulated with PMA (100 nM) or infected with ZIKV (MOI 2) for 6 hours in 8-chamber culture. Cells were stained for DNA with Hoechst 33342. Scale bars = 100 μm, Magnification = 20×. (**D**,**E**) Neutrophils (2 × 10^6^/mL) were stimulated with ZIKV, UV-ZIKV or PMA or left unstimulated for 6 hours. Extracellular DNA (μg/mL) was quantified in culture supernatants and fold number of concentrations was evaluated using the negative control (NCrl) as reference. Data are representative of 2 independent experiments performed in triplicates and represent mean ± SD. Data were analyzed with Kruskal-Wallis test. *p < 0.05, **p < 0.01.

Among the biomarkers characterized in this study, we identified a particular class of endogenous metabolites of the lipoxygenase system, hepoxilins, namely two isomers: hepoxilins A_3_ and B_3_ (HxA_3_ and B_3_). These species have been described in the literature as signaling molecules associated with post-injury axonal repair mechanisms^29^, promotion of neurite regeneration^30^, as well as inducers of neutrophil extracellular traps (NETs)^31^. As ZIKV is able to trigger innate immune response in different cells types, such as dendritic cells, monocytes, and lymphocytes^32^, we decided to test whether ZIKV infection might induce NETs formation in human neutrophils *in vitro*. For this, we monitored the presence and concentration of extracellular DNA in human neutrophils after four treatments: a positive control (PMA), when NETs were induced by phorbol myristate acetate, a negative control (NCtrl), and when cells were infected with wild-type ZIKV or inactivated ZIKV by ultraviolet for 30 minutes (UV-ZIKV). As demonstrated in Fig. 3, extracellular DNA is not significantly increased in ZIKV-infected neutrophils after 6 hours post-infection. Thus, we hypothesize that hepoxilins are specifically acting as anti-inflammatory mediators in the aftermath of neural depletion, attempting to minimize the overall damage (Fig. 2). Furthermore, there is clinical evidence that ZIKV remains in the CNS even after birth, as Chimelli, *et al*.^33^ reported a case of ZIKV persistence after birth in an infant with CZS, evidenced by viral RNA presence in the urine at birth, and in the brain in postmortem tissue analyses. The findings from Arruza, *et al*.^34^ also provide support to the idea that neural damage provokes an integrated response, i.e. the effects and metabolites associated with inflammation may be present even in tissues that are distant from the actual threat. All these evidences are complementary, and consistent, therefore, with a condition of persistent neuroinflammation, in which hepoxilins and other prostaglandin derivatives are trying to counterbalance the action with anti-inflammatory processes.

Our contention is that the broad spectrum of symptoms associated with congenital Zika syndrome affecting infants born to mothers infected with ZIKV are the result of viral induced microglial activation and excitotoxicity. The presence of the anti-inflammatory biomarkers in the saliva, even for longer periods after birth, indicate that the immune system remains active for an indefinite period. Therefore, in addition to the monitoring of cognitive development, which is the main focus of current public health policies for infants born with CZS, inflammation status and its relationship with the persistence of ZIKV after birth should also be investigated. Thus short- and long-term outcomes associated with ZIKV syndrome should be investigated using selected cohorts of CZS patients, especially at the molecular level, with care taken to control for other congenital infections which may additionally affect CNS development and/or elicit inflammatory responses. Finally, saliva emerges as a viable, non-invasive alternative to assess neural diseases, which is coherent due to the interactions between salivary glands and developing nerves; the use of this biofluid in prospective metabolomics studies is a current trend, and is being further explored in studies related to conditions that affect the nervous system^35^.

## Methods

### Metabolomics of saliva

#### Patient selection

Twenty-seven infants diagnosed with CZS according to an outlined phenotypic spectrum by neuroimaging^36^ were selected to participate in this study, which is approved by the Brazilian Research Ethics Committee under the number 61936216.9.0000.5404. The counterpart was a control group, composed of healthy individuals – 27 infants with no clinical signs and symptoms, nor neuroimaging diagnosis of CZS. Gender was not matched between groups, and the age limit for any group was 2 years of age; the choice to not homogenize groups was so that we would be able to assess the specificity of the proposed model in identifying disease-related molecules of interest. All experiments were performed in accordance with the guidelines provided by the Declaration of Helsinki. Since all individuals were under the age of 18 years, the written informed consent was obtained from their parents and/or legal guardians for sample collection and use within the scope of this research.

#### Sample collection and high-resolution mass spectrometry

Saliva samples were collected using the Oragene^®^ DNA Self-Collection Kit (DNA Genotek Inc., Ontario, Canada) as per the manufacturer’s instructions. Samples were maintained frozen under −80 °C until analysis. 10 microliters of saliva were diluted in a methanol/water solution (1:1) to a final volume of 1 mL. The solution was homogenized under vortex, filtered through a PVDF membrane (0.22 µm); formic acid or ammonium hydroxide were added to a concentration of 0.1% to assist positive and negative ion formation, respectively. Final solutions were sent for analysis by high resolution mass spectrometry (ESI-LTQ-XL Orbitrap Discovery, Thermo Scientific, Bremen, Germany) both in positive and negative ion modes, in the mass range of 100–1000 *m/z*. Markers for the CZS group were determined with the assistance of online metabolomics platform METLIN (http://metlin.scripps.edu).

#### Statistical analysis

Spectrometric data were submitted to multivariate statistical analysis using Orthogonal Partial Least Squares Discriminant Analysis (OPLS-DA) in the online metabolomics-processing platform MetaboAnalyst^37^. Data were normalized with reference to the CZS group, with range scaling. Most relevant features elected by the variable importance in projection (VIP) scores were selected. One-hundred cross-validations (CV) were performed, and prediction accuracy during training was calculated using 1000 permutations for data from both ion modes (p = 0.001, data not shown) to assess significance and validate the proposed statistical model.

### Assessment of NETs *in vitro* after neutrophil induction by ZIKV

#### Reagents

MEM (Minimum Essential Medium) and Dextran from *Leuconostoc* spp. were purchased from Sigma-Aldrich (St. Louis, MO). Qubit dsDNA HS assay kit was from Invitrogen (Carlsbad, CA). Ficoll-Paque PLUS was from GE Healthcare (Chicago, IL).

#### Human neutrophil isolation

Whole blood (20 mL) was collected from healthy volunteer donor into heparin-treated tubes. Neutrophils were purified by density gradient centrifugation using Ficoll-Paque PLUS (GE Healthcare, Chicago, IL)^38^. Erythrocytes were removed by dextran sedimentation followed by two rounds of hypotonic lysis. Purified neutrophils were re-suspended in MEM medium (Sigma-Aldrich, St. Louis, MO).

#### Stimulation of neutrophils and quantification of extracellular DNA

Neutrophils (2 × 10^6^ cells/mL) were stimulated with PMA (100 nM), ZIKV or UV-inactivated ZIKV at an MOI of 2 for 6 hours at 37 °C under 5% CO_2_. After the stimulation period, culture supernatant was collected, and extracellular DNA was precipitated using 3 M sodium acetate and ethanol; the obtained pellet was then washed with 70% ethanol and resuspended in nuclease-free water. Isolated DNA samples were measured using either Quant-iT dsDNA or Qubit dsDNA HS kits (Invitrogen, Carlsbad, CA), following manufacturer’s instructions. The ZIKV strain was obtained and maintained according to the procedures by Melo *et al*.^39^.

#### Immunofluorescence

Neutrophils (1 × 10^5^/300 μL) were seeded in 8-chamber culture slides and incubated with PMA (100 nM) or ZIKV (MOI 2) for 6 hours at 37 °C under 5% CO_2_. Cells fixed with 4% paraformaldehyde (PFA) and were stained with Hoechst 33342 (1:1000). Images were taken using 20x magnification in Cytation 5 (BioTek, Vermont).

#### Statistical analyses

Data were presented as mean ± SD. The results obtained were analyzed using GraphPad Prism (version 6.0, GraphPad Software, Inc., San Diego, CA) statistical software package. Comparisons between multiple groups were analyzed using nonparametric test with a posteriori Kruskal-Wallis test. The level of significance was set at p ≤ 0.05.
